# Plasma CXCL10, sCD163 and sCD14 Levels Have Distinct Associations with Antiretroviral Treatment and Cardiovascular Disease Risk Factors

**DOI:** 10.1371/journal.pone.0158169

**Published:** 2016-06-29

**Authors:** Alison Castley, Leah Williams, Ian James, George Guelfi, Cassandra Berry, David Nolan

**Affiliations:** 1 PathWest Laboratory Medicine, Department of Clinical Immunology: Royal Perth Hospital, Perth, Western Australia, Australia; 2 School of Veterinary and Life Sciences, Murdoch University, Perth, Western Australia, Australia; 3 Institute for Immunology and Infectious Diseases (IIID), Murdoch University, Perth, Western Australia, Australia; University of Pittsburgh Center for Vaccine Research, UNITED STATES

## Abstract

We investigate the associations of three established plasma biomarkers in the context of HIV and treatment-related variables including a comprehensive cardiovascular disease risk assessment, within a large ambulatory HIV cohort. Patients were recruited in 2010 to form the Royal Perth Hospital HIV/CVD risk cohort. Plasma sCD14, sCD163 and CXCL10 levels were measured in 475 consecutive patients with documented CVD risk (age, ethnicity, gender, smoking, blood pressure, BMI, fasting metabolic profile) and HIV treatment history including immunological/virological outcomes. The biomarkers assessed showed distinct associations with virological response: CXCL10 strongly correlated with HIV-1 RNA (p<0.001), sCD163 was significantly reduced among ‘aviraemic’ patients only (p = 0.02), while sCD14 was unaffected by virological status under 10,000 copies/mL (p>0.2). Associations between higher sCD163 and protease inhibitor therapy (p = 0.05) and lower sCD14 with integrase inhibitor therapy (p = 0.02) were observed. Levels of sCD163 were also associated with CVD risk factors (age, ethnicity, HDL, BMI), with a favourable influence of Framingham score <10% (p = 0.04). Soluble CD14 levels were higher among smokers (p = 0.002), with no effect of other CVD risk factors, except age (p = 0.045). Our findings confirm CXCL10, sCD163 and sCD14 have distinct associations with different aspects of HIV infection and treatment. Levels of CXCL10 correlated with routinely monitored variables, sCD163 levels reflect a deeper level of virological suppression and influence of CVD risk factors, while sCD14 levels were not associated with routinely monitored variables, with evidence of specific effects of smoking and integrase inhibitor therapy warranting further investigation.

## Introduction

In spite of the evident success of highly active antiretroviral therapy (HAART) in suppressing plasma levels of HIV-1 RNA, preventing progressive immune deficiency and ultimately improving patient survival [1], there is increasing evidence that immune activation persists in the face of effective HIV treatment [2,3]. This immune phenotype, which is characterized by prominent monocyte activation [2–4], heightens age related changes [5] and has been associated with increased prevalence and earlier onset of a range of non-infectious co-morbidities among HIV-infected individuals including cardiovascular and liver disease, type II diabetes mellitus and cognitive decline [2–4,6]. The prognostic significance of several innate immune ‘biomarkers’ such as interleukin-6, C-reactive protein and particularly soluble CD14 (sCD14) [7] as strong predictors of mortality in the setting of treated HIV infection, has also now been established [2–4].

We have previously described an ongoing systemic inflammatory response to HIV infection among 81 HIV+ individuals with a range of treatment outcomes compared to 21 healthy control blood donors [8]. Here, untreated HIV infection was characterised by elevated levels of pro-inflammatory CD16^+^ monocytes as well as elevated plasma levels of monocyte-derived, interferon-inducible proteins (sCD14, soluble CD163 and CXCL10). Treatment-associated suppression of plasma HIV-1 RNA levels (i.e. <40 copies/mL) was associated with levels of sCD163 and CXCL10 that were similar to healthy controls, while sCD14 levels remained significantly elevated despite what would otherwise be considered successful HIV treatment [8]. This observation of stable elevated sCD14 levels has also been made by others [9,10], highlighting that plasma ‘biomarkers’ of systemic immune activation have distinct relationships with HIV infection and its treatment.

In this study we have sought to investigate these plasma ‘biomarkers’ further, utilising a larger study population and incorporating analysis of cardiovascular disease risk factors, noting that sCD14 has been positively correlated with smoking, diabetes, fasting glucose and hypertension (all p<0.001), as well as all-cause mortality, in a large US cohort study of older adults [11]. We were also interested to explore the influence of detectable plasma HIV-1 RNA below the standard viral load assay threshold of 40 copies/mL on these biomarkers, having previously demonstrated in a study of >11,000 viral load results that ‘residual viraemia’ could be identified in 20% of samples measured at <40 copies/mL and was strongly predicted by the level of plasma viraemia prior to HIV treatment–even after 10–15 years of suppressive HIV therapy [12].

Our principal aim was to investigate the potential utility of incorporating one or more of these plasma biomarkers into routine HIV management, through an improved understanding of their relationships to known laboratory and clinical variables. This is informed by a growing awareness that monitoring CD4^+^ T cell counts has limited ongoing utility once normal levels have been achieved [13], while other markers of immune function may have more prognostic value [7] as well as providing insights into disease pathogenesis [14] and informing new therapeutic strategies beyond the current antiretroviral treatment paradigm [15].

## Materials and Methods

### Patient cohort

Patients residing in Western Australia and attending the Royal Perth Hospital (RPH) Immunology clinic in 2010 were recruited for this study. Informed written consent was obtained from the patients participating in this investigation. This consent form was reviewed and accepted by the ethics committees. Written ethics committee approvals for this investigation were received from Royal Perth Hospital (EC2012/170) and Murdoch University (2012/216).

### Plasma collection process

Plasma samples were collected on the day of CVD risk assessment. Plasma was collected from EDTA whole blood samples within 6 hours of collection by centrifugal force of 1000g for 20 mins. Plasma was removed and stored at -80°C until required for the plasma HIV-1 RNA viral load and enzyme-linked immunosorbent assays (ELISA).

### Cardiovascular disease risk assessments and HIV laboratory testing

Data on cardiovascular disease risk factors were obtained by physical examination and blood tests assessed at the time of visit. Serum levels of total cholesterol, high density lipoprotein (HDL), low density lipoprotein (LDL) and triglycerides were measured and the total:HDL ratio was subsequently calculated.

HIV-1 RNA levels were measured using the Roche Cobas ultrasensitive ampliprep assay V1 (Roche). CD4^+^ T cell counts, CD4^+^ T cell percentage and CD4:8 ratios were measured from sample acquisitions performed on the FACSCanto II flow cytometer with FACSDiva 6.1.1 software (BD Biosciences).

Physical examinations included records for age (at sample collection), gender, ethnicity, height (cm), weight (kg) and blood pressure monitoring (FsysBP, FDiaBP). Smoking was self-reported and recorded as either being a smoker, a non-smoker or an ex-smoker (within 1 year). Clinical notes were accessed to record whether patients were using ART (protease inhibitor, NRTI, NNRTI and Integrase inhibitor), ACE inhibitors or statin therapy at the time of assessment. Body mass index (BMI) was calculated using height and weight measurements overseen by a dietitian, whilst the Framingham score (mean 5-year CVD risk score) was determined using the National Heart Foundation absolute CVD risk algorithm.

### Measurement of plasma sCD14, sCD163 and CXCL10 levels

For the quantitative determination of plasma biomarker levels, ELISAs were utilised, without modification, as previously described [7]. In-house control samples (sCD14 = 400,000 ρg/μl; sCD163 = 250 ηg/μl; CXCL10 = 125 ρg/μl) were included in each assay. The mean concentration values (SD) for the assay controls over all runs were 444,080 ρg/μl (17310), 251 ηg/μl (16.7) and 120.8 ρg/μl (5.76) for sCD14, sCD163 and CXCL10 respectively. The mean R value from all ELISAs was 0.993, 0.997 and 0.998 for sCD14, sCD163 and CXCL10 respectively.

### Statistical analysis

Statistical data analysis was performed using SPSS version 21. Data distribution was assessed for normality, with transformation of variables as required. Plasma biomarker data required logarithmic transformation while absolute CD4^+^ cell count, CD4:8 ratio and total cholesterol levels were square rooted. One way ANOVA analysis was utilised to compare variants within the HIV cohort with appropriate correction for multiple comparisons when >2 groups were compared. Univariate correlation and multivariate linear regression analyses were utilised to estimate associations between plasma biomarkers and HIV clinical parameters (viral load status, HIV-1 RNA viral load level, absolute CD4^+^ T cell count, CD4:8 ratio), treatment choice, CVD risk factors (blood pressure and cholesterol variables, smoking, BMI and Framingham score), gender, age and ethnicity. Statistical significance required a p-value of <0.05.

## Results

The study population included 474 consecutive patients who attended the Royal Perth Hospital (RPH) Immunology clinic in 2010 who consented to a cardiovascular risk assessment including smoking history, standardised measurements of blood pressure and weight as well as collection of a fasting metabolic profile. Patient characteristics, demographics and clinical details for this study are shown in Table 1.

**Table 1:** Demographics and patient characteristics from 474 HIV positive patients who underwent CVD risk assessments in 2010.

*Characteristic*	*Value*
Age at time of assessment, mean years (range)	45 (21–81)
Male sex (n, %)	372 (78.5)
Ethnicity	
- Caucasian (n, %)	326 (68.8)
- Indigenous Australian (n, %)	24 (5.1)
- African (n, %)	57 (12.1)
- Asian (n, %)	66 (14.0)
Current Smoker (n, %)	168 (35.4)
HIV Clinical Parameters at time of assessment	
- Plasma HIV RNA viral load (lcpm)	2.4 (1.6–6.0)
- Aviraemia (n, %)	211 (44.5)
- Residual viraemia <1.6 lcpm (n, %)	60 (12.7)
- HIV RNA viral load 1.6-≤3 lcpm (n, %)	75 (15.8)
- HIV RNA viral load 3–4 lcpm (n, %)	49 (10.3)
- HIV RNA viral load ≥4 lcpm (n, %)	79 (16.7)
- CD4%, mean % (range, SD)	26.1 (1–62, 11)
- Absolute CD4 T cell count, mean (range, SD)	567 (3–2205, 319)
- CD4:8 ratio, mean (range, SD)	0.65 (0.01–3.1, 0.4)
HIV therapy at the time of assessment (n)	365
- NNRTI (n, %)	215 (45.4)
- NRTI (n, %)	348 (73.4)
- HIV Protease Inhibitor (n, %)	161 (34)
- Integrase (n, %)	17 (3.6)
Framingham score (mean, range)	6.67 (0–42)
BMI, kg/m^2^ (mean, SD)	25 (4.8)
Statin therapy, n (%)	72 (15.2)
Diabetic, n (%)	25 (5.3)
ACE inhibitor, n (%)	54 (11.4)
sCD14 (log ρg/μl), mean (SD error, range)	6.24 (0.007, 5.65–6.62)
sCD163 (log ηg/μl), mean (SD error, range)	2.89 (0.009, 2.22–3.42)
CXCL10 (log ρg/μl), mean (SD error, range)	2.05 (0.018, 1.13–3.22)

lcpm = log copies per mL, SD error = standard error; n = number, BMI; body mass index; ACE = angiotensin converting-enzyme inhibitor; NRTI = Nucleoside Reverse Transcriptase; NNRTI = Non Nucleoside/Nucleotide Reverse Transcriptase.

The overall study population comprised 78.5% males, 68.8% Caucasians, with a mean age of 45 years (range 21–81 years, SD 12.3 years). The overall mean CD4^+^ T cell count was 567 cells/μL (range 3–2205 cells/μL, SD 319 cells/μL) and mean plasma HIV-1 RNA level was 2.4 log_10_copies/mL (lcpm) or 251 copies/mL (cpm). Three hundred and sixty-five patients were on antiretroviral therapy (77%), with undetectable HIV-1 RNA viral load (<40 cpm) noted for 271 patients; 57.2% of the overall cohort, and 74.2% of those on HIV therapy. Within this subset, target HIV-1 amplification could be detected on the Cobas HIV-1 ampliprep system below the assay threshold of 40 cpm, reflecting ‘residual viraemia’ in 60 cases (22.1% of results reported as <40 cpm).

With regard to cardiovascular risk factors, the mean 5-year CVD risk score (Framingham score) in the study population was 6.6% (SD 6.7; range 0–42), including 325 patients (71.0%) in the low risk category (estimated 5-yr CVD risk <10%), 90 patients (19.7%) deemed at moderate risk (5-yr CVD risk 10–15%), and 43 patients within the high risk group (n = 23 (5.0%) with CVD risk 15–20%, and n = 20 (4.4%) with CVD risk >20%). The study group included 168 current smokers (35.4%) and 25 diabetics (5.3%). The average BMI of the study group was 25.0 kg/m^2^ (SD 4.8). Statin therapy was used in 15.2% while ACE inhibitors were used in 11.4% of participants.

Plasma biomarker levels were approximately normally distributed following logarithmic transformation, with mean log values and corresponding plasma concentrations for sCD14, sCD163 and CXCL10 of 6.24 [1,737,800 ρg/μl], 2.89 [776 ηg/μl] and 2.05 [112.2 ρg/μl] respectively (Table 1).

### Correlations between HIV-1 clinical parameters, CVD risk and circulating plasma biomarkers

**Fig 1:**
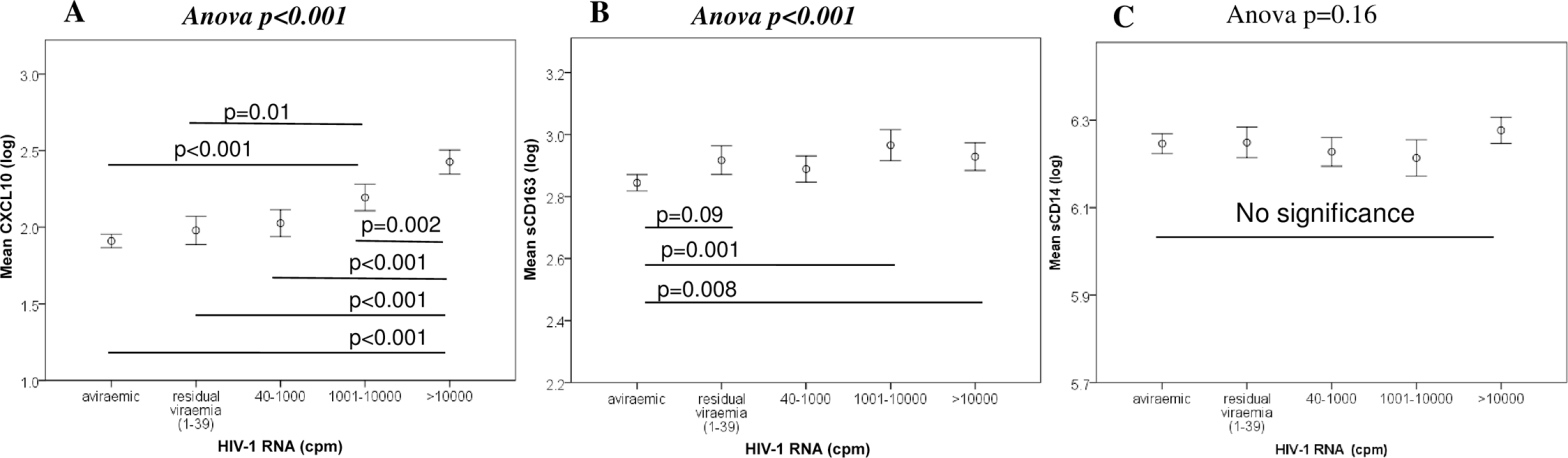
Differing correlation outcomes between the three plasma biomarkers and HIV-1 RNA levels. A significant correlation was recognised between HIV-1 RNA levels with CXCL10 (A) and sCD163 (B) while there was no significance with sCD14 (C).

Spearman rank correlations were used to explore associations between circulating soluble plasma biomarkers and HIV clinical and CVD risk parameters. Investigating the influence of HIV clinical parameters (HIV-1 RNA, CD4^+^ T cell count and CD4:8 ratio), we observed strong positive correlations between plasma HIV-1 RNA levels and both CXCL10 and sCD163 (p<0.001, r = 0.5: Fig 1A and 1B) and strong negative correlations for CD4^+^ T cell (p<0.001, r = -0.36) and CD4:8 ratio (p<0.001, r = -0.37) while we could not find any significant correlations between HIV clinical parameters and sCD14 (p>0.1, Fig 1C).

**Fig 2:**
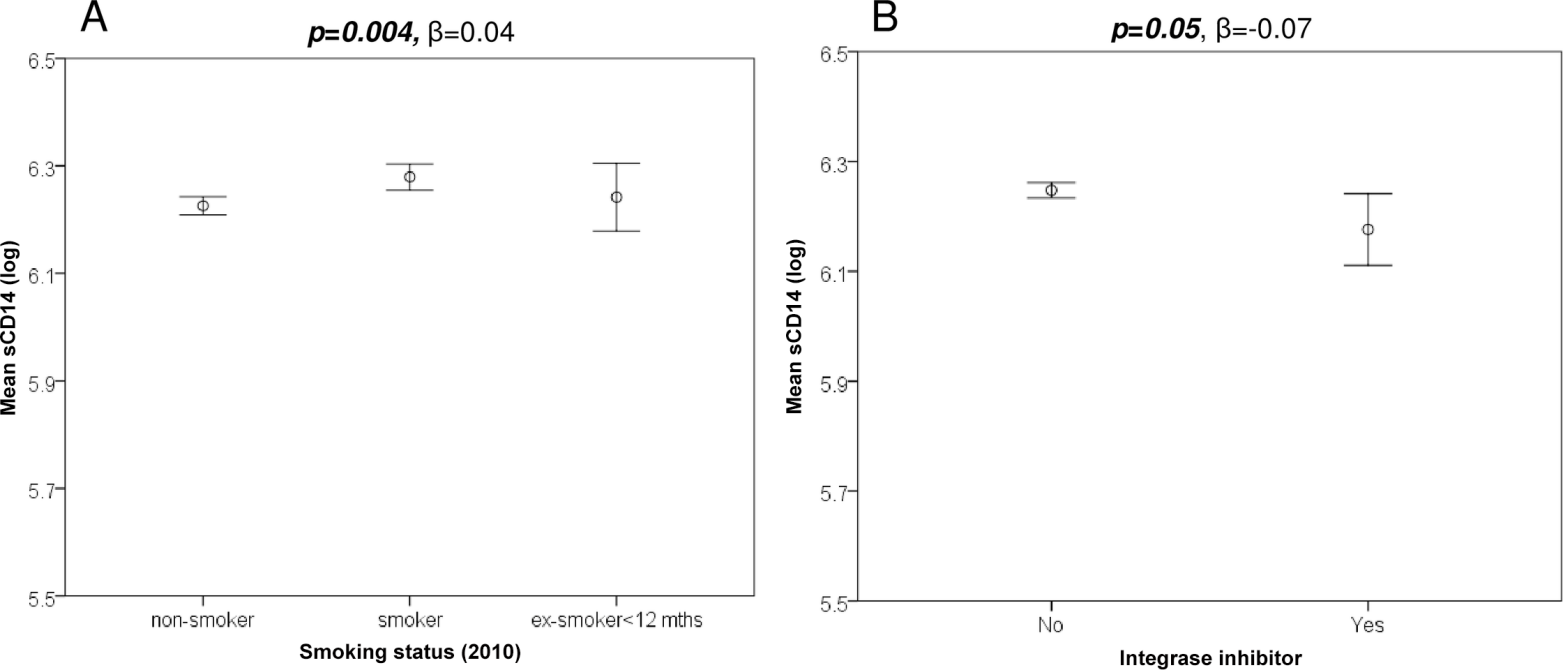
There was a significantly strong correlation between sCD14 and smoking where HIV-1 smokers have higher sCD14 levels than HIV-1 non-smokers while patients on an integrase inhibitor had significantly lower sCD14 levels than patients on an alternative treatment.

With respect to CVD risk, we demonstrated a strong and significant negative correlation for sCD163 and CXCL10 and total cholesterol levels (data not shown; p<0.001, r = -0.23), LDL-c (p<0.01, r = -0.18) and HDL-c (p≤0.001, r = -0.18), however these parameters did not correlate with sCD14 levels (p>0.6). There were no significant correlations between the plasma biomarkers and total:HDL cholesterol ratio (p>0.1), blood pressure parameters (p>0.2), Framingham score (p>0.15) or BMI (p>0.07). Analysis with the Tukey post-hoc test revealed lower sCD14 levels in non-smokers compared to smokers (Fig 2A: p = 0.004). Furthermore, there was a significant positive correlation for sCD14 and sCD163 levels with smoking (data not shown; p = 0.002, r = 0.14; p = 0.03, r = 0.1 respectively) but not CXCL10 (p = 0.07).

We also demonstrated correlations between the three plasma biomarkers assessed. Circulating sCD163 had a strong positive correlation with CXCL10 levels (p<0.001, r = 0.41, S1A Fig) and sCD14 levels (p<0.001, r = 0.17, S1B Fig) however sCD14 did not significantly correlate with CXCL10 (p = 0.07, r = 0.07 S1C Fig). We did not identify any correlations for age (p>0.09) or gender (p>0.4) with circulating plasma biomarkers. Univariate analysis of the plasma biomarkers with ethnicity suggests higher levels of CXCL10 and sCD163 in Indigenous Australians (S2A and S2B Fig) and lower levels of sCD14in Africans (S2C Fig).

### Multivariate regression analysis reveals distinct biomarker associations

Multivariate regression analysis was then undertaken with each plasma biomarker in isolation (Model 1) as well as considering the influence of all three biomarkers in adjusted analyses (Model 2).

#### CXCL10

In unadjusted analyses for Model 1 (S1 Table), CXCL10 levels were strongly associated with higher HIV-1 RNA viral load (p<0.0001) and lower CD4^+^ T cell counts (p = 0.0001) as well as lower CD4:8 ratio (p = 0.008). Participants on NRTI therapy had lower CXCL10 (p = 0.0002) whilst being an Asian (p = 0.05) or African male had favourable effect on CXCL10 levels (p = 0.0001, β>0.2). Framingham score and BMI were not associated with CXCL10 levels, however patients on an ACE inhibitor had significantly higher levels of CXCL10 than patients not on an ACE inhibitor (p = 0.045, β = 0.1). Lower total cholesterol levels were also associated with elevated CXCL10 levels (p = 0.02, β = -0.37).

As shown in Table 2 (Model 2), the inclusion of all plasma biomarkers did not abrogate the significant associations of CXCL10 with HIV clinical parameters (CD4^+^ T cell counts (p = 0.0005) and CD4:8 ratio (p = 0.012) and HIV-1 RNA viral load (p<0.0001), ethnicity (p = 0.001), NRTI therapy (p<0.001) or with sCD163 (p<0.0001). It did however nullify the significant association with ACE inhibitor treatment and cholesterol levels. Interestingly, the adjusted multivariate regression analysis confirmed an interaction between gender and ethnicity (Table 2) which attributed to significantly lower CXCL10 levels in Asian (p = 0.001, β = -0.15) and African males (p<0.001, β = -0.22).

**Table 2:** Model 2—Multivariate regression results showing significant associations of plasma biomarker with HIV clinical parameters, CVD risk age, gender, ethnicity and smoking after adjusting for CXCL10, sCD163 and sCD14.

*Biomarker*	*CXCL10*			*sCD163*			*sCD14*		
*Variable*	β	std error	p	β	std error	p	β	std error	p
Age at 2010	-	-	-	0.002	0.001	***0*.*03***	0.001	0.0006	0.08
Gender	-	-	-	-	-	-	0.031	0.018	0.08
Ethnicity	-0.15	0.046	***0*.*001***	0.107	0.042	***0*.*012***	-0.058	0.022	***0*.*009***
Gender:Ethnicity	-0.224	0.063	***0*.*0004***	-	-	-	-	-	-
Smoking	-	-	-	-	-	-	0.043	0.015	***0*.*004***
Residual viraemia	-	-	-	0.058	0.026	***0*.*025***	-	-	-
VL5	0.28	0.058	***<0*.*0001***	-	-	-	0.07	0.023	***0*.*002***
SQR CD4:8	-0.202	0.080	***0*.*012***	-	-	-	-	-	-
SQR CD4	-0.01	0.003	***0*.*0005***	-	-	-	-0.002	0.001	0.07
NRTI	-0.156	0.046	***0*.*0007***	-	-	-	-	-	-
NNRTI	-	-	-	-	-	-	0.052	0.018	***0*.*004***
PI	-	-	-	-	-	-	0.041	0.018	***0*.*026***
Integrase	-	-	-	-	-	-	-0.078	0.037	***0*.*037***
HDL	-	-	-	-0.04	0.023	*0*.*09*	-	-	-
BMI	-	-	-	0.004	0.002	***0*.*017***	-	-	-
Framingham score	-	-	-	0.05	0.025	***0*.*049***	-	-	-
CXCL10	-	-	-	0.18	0.026	***<0*.*0001***	-0.023	0.022	0.3
sCD163	0.51	0.079	***<0*.*0001***	-	-	-	0.13	0.039	***0*.*0009***
sCD14	-0.06	0.096	0.53	0.16	0.056	***0*.*003***	-	-	-

“-“p = >0.01; std = standard error.

#### sCD163

In unadjusted analysis plasma levels of sCD163 were significantly reduced among ‘aviraemic’ patients only (p = 0.02, β = 0.06) and otherwise remained stably elevated across all levels of virological suppression (Model 1; S1 Table). Higher levels were associated with lower CD4^+^ T cell counts (p = 0.01, β = -0.004), as previously noted for CXCL10. Several CVD risk factors were associated with sCD163, namely age (p = 0.001, β = 0.003), ethnicity (p = 0.01, β = 0.1), HDL (p = 0.048, β = -0.05) and BMI (p = 0.009, β = 0.005), with a favourable influence of Framingham score <10% (p = 0.04, β = 0.06). Additionally, the level of circulating sCD163 was increased when the choice of HIV treatment was a PI (p = 0.05, β = 0.04) but decreased if participants were on NRTIs (p = 0.04, β = -0.06).

Including all the biomarkers in the regression analysis for sCD163 (Model 2; Table 2) showed a positive association with both CXCL10 (p<0.0001) and sCD14 (p = 0.003). Interestingly sCD163 remained significantly associated with ‘aviraemic’ status only (p = 0.02) and with several CVD risk factors including age (p = 0.03), ethnicity (p = 0.01), BMI (p = 0.02), Framingham score <10% (p<0.05). Univariate associations with CD4^+^ T cell counts and choice of therapy were abrogated in adjusted analyses. Of interest, the adjusted multivariate regression analysis confirmed that sCD163 levels were significantly higher among indigenous cases (p = 0.012, β = 0.11).

#### sCD14

Analysing the determinants of sCD14 levels in Model 1 (S1 Table) revealed no significant influence of virological status under 10,000 cpm (p = 0.8: data not shown) although sCD14 levels were incrementally higher when HIV-1 RNA levels were >10,000 cpm (p = 0.003, β = 0.06). Soluble CD14 levels were higher among smokers (p = 0.002, β = 0.05), lower in Africans (p = 0.009, β = -0.06) with no effect of other CVD risk factors or overall Framingham score, apart from age (p = 0.04, β = 0.001). Interestingly, lower sCD14 levels were associated with use of integrase inhibitor therapy (p = 0.02, β = -0.09; also Fig 2B).

After adjusting for soluble biomarkers in Model 2 (Table 2), sCD14 levels remained strongly associated with sCD163 (p<0.001), HIV-1 RNA level >10,000 copies/mL(p = 0.002), smoking (p = 0.004), ethnicity (p = 0.009) and choice of HIV treatment–with a beneficial effect of integrase inhibitor therapy (p = 0.037). In this analysis, the univariate association with age was abrogated (p = 0.08). Interestingly, the adjusted multivariate regression analysis confirmed that sCD14 levels were significantly lower for African cases (p = 0.009, β = -0.06).

## Discussion

**Fig 3:**
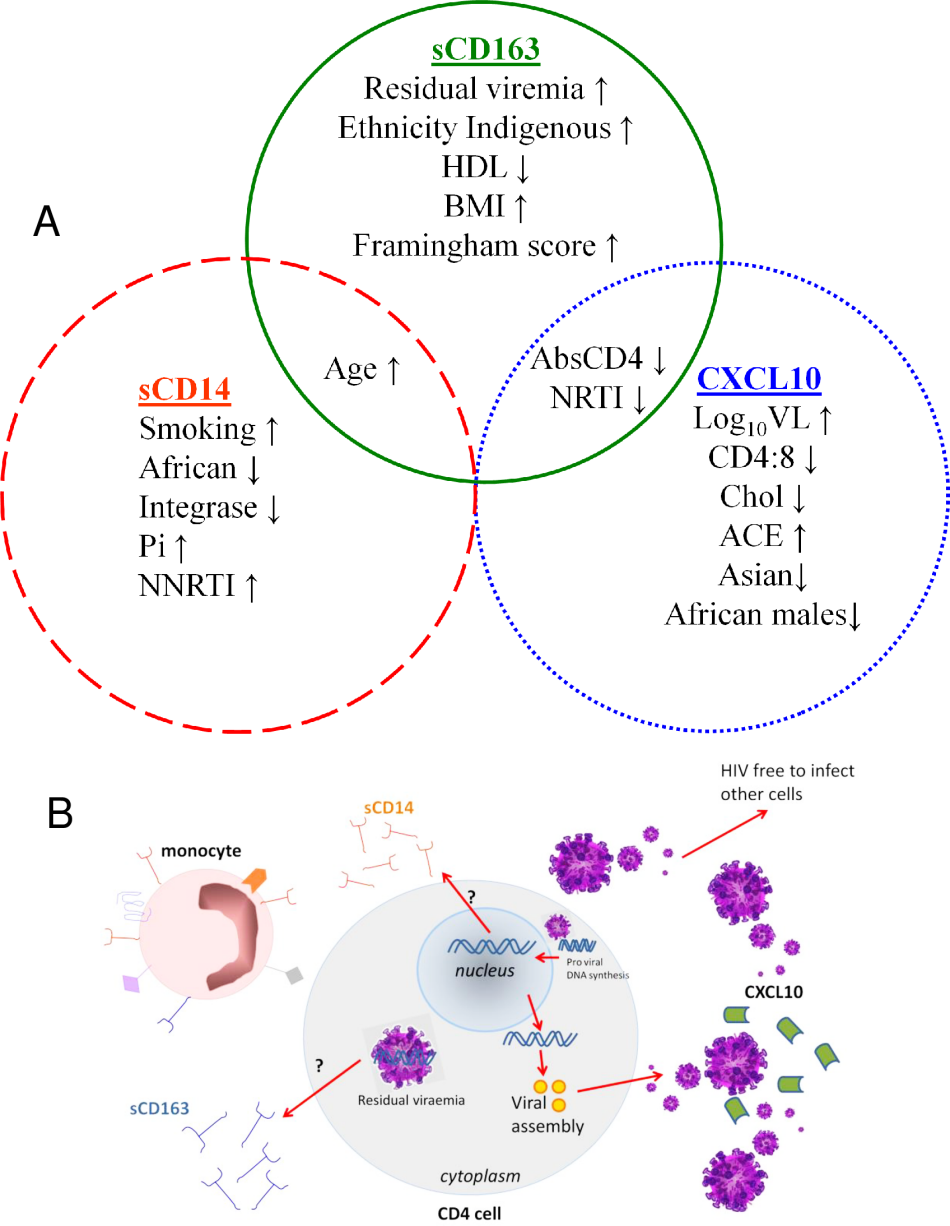
CXCL10, soluble CD14 and soluble CD163 have distinct although overlapping associations with cardiovascular disease risk factors, HIV treatment, ethnicity and age (A) along with different aspects of HIV infection (B).

This study confirms our previous finding that CXCL10, sCD163 and sCD14 have distinct although overlapping associations with different aspects of HIV infection and treatment [8], as well as cardiovascular disease risk factors and demographic variables (Fig 3). These relationships are in keeping with an increasingly refined understanding of these plasma biomarkers and their place within the immune environment. For example, CXCL10 was initially identified as an interferon-gamma-induced protein (also named interferon inducible protein-10) and a ligand for CXCR3 [16,17], although in the context of HIV infection there is evidence that the strong relationship between plasma viraemia and CXCL10 [5, 8–10,18] is likely to be mediated via IFN-α-induced toll-like receptors 7 and 8 [18]. Circulating plasma virions therefore provide the major stimulus for CXCL10 secretion from monocytes and monocyte-derived dendritic cells [18], which in turn inhibits IFN-γ signalling and adaptive immune responses [19]. In this respect, CXCL10 appears to have its most important prognostic role in early HIV infection, where elevated levels independently predict disease progression rate [20] even among HIV-controllers with low levels of viraemia [21], and are also associated with risk of transmitting or acquiring HIV infection [22]. In this setting, the potential utility of CXCL10 measurement in clinical practice is likely to diminish in light of recent evidence that early treatment of HIV infection is beneficial irrespective of baseline CD4^+^ T cell count or plasma viral load [1].

Soluble CD163 provides an interesting contrast in that elevated plasma levels are associated with a range of cardiovascular risk factors (age, HDL cholesterol, body mass index), and perhaps most importantly with the overall Framingham cardiovascular risk score. This is in keeping with a number of studies that have identified associations between sCD163 and cardiovascular inflammation and atherosclerotic plaque formation in the setting of HIV infection [14,23–25] as well as in the general population [23,26,27]. This association appears to be underpinned by the role of activated monocyte-derived macrophages within atherosclerotic vessels [28] as well as in adipose tissue [29,30] in producing sCD163. This pathway involves tumor necrosis factor-alpha (TNF-α) and ADAM-17 [30,31], thus providing a link between inflammation and atherosclerotic risk factors including oxidised lipids that may be particularly relevant in HIV infection [32,33] but are rarely measured in routine care.

We previously found that sCD163 levels were elevated in the setting of untreated HIV infection, while levels among those on suppressive antiretroviral therapy were comparable to healthy controls [8]. Here we extend this observation, noting that sCD163 levels were significantly reduced only among those patients with no detectable plasma HIV-1 RNA, and remained relatively elevated in the 60 cases with residual viraemia (22.1% of results reported as <40 cpm). This is consistent with previous studies that have identified the TNF-α pathway as a sensor of low-level viraemia [34,35], although to our knowledge sCD163 has not been previously studied in this context. Given previous evidence that persistent low-level viraemia originates largely from a reservoir of long-lived, latently-infected CD4^+^ T cells [36,37], it is interesting to note that ADAM-17 and TNF-α (the major stimuli for sCD163 shedding [31]) are implicated in the replication of quiescent CD4^+^ T lymphocytes initiated by exosomes from HIV Nef-expressing cells [38].

These findings suggest that measuring soluble CD163 in routine HIV care has the potential to capture important prognostic information regarding cardiovascular risk [14,23–25] and other co-morbid conditions [39] as well as HIV treatment responses beyond the detection threshold of routine viral load measurements.

The measurement of soluble CD14 in this study population confirmed previous observations by ourselves [8] and others [9,10,40,41] that levels remain elevated irrespective of the level of plasma HIV suppression. In contrast to the broad influence of cardiovascular risk factors on sCD14 levels in the general population [11], we did not observe any influence of individual risk factors or the overall Framingham score–suggesting that HIV infection itself provided an overriding stimulus. We did however observe a significant influence of cigarette smoking, which was not examined specifically in the population-based study but has been identified previously in the setting of HIV infection [42]. Moreover, we identified a favourable effect of integrase inhibitor-based HIV treatment on sCD14 levels in this study, despite the small number of patients receiving this regimen in 2010 (n = 17). This effect has also been observed by others [43,44] although not universally [40,45], and at this stage an underlying mechanism has not been identified although an association between sCD14 and integrated HIV DNA has been observed in one study [46]. This warrants further study, along with the impact of smoking, particularly given the established mortality risk associated with elevated sCD14 levels [7] as well as the ongoing strong influence of smoking on mortality among HIV^+^ individuals [47,48].

The major strength of this analysis is the statistical power associated with a large sample pool with complete HIV treatment history and full CVD risk assessments. Limitations include the lack of subclinical measurements of vascular disease, so that we were unable to confirm previous findings of associations between sCD14 or sCD163 and cardiovascular disease or mortality. This study lacked an HIV negative control group, however, we have previously provided evidence of significantly lower sCD14 in an HIV negative population compared to treated and untreated HIV groups, while sCD163 and CXCL10 were reduced to levels seen in HIV uninfected controls when treated with antiretroviral therapy [8].

In summary, this study supports growing evidence that monitoring plasma immune activation markers has prognostic significance throughout the course of HIV management. We provide further evidence that these biomarkers, although linked through common associations with monocyte activation and interferon signalling, reveal distinct aspects of the inflammatory response and indeed of the HIV replication cycle. We found that levels of CXCL10 correlated with routinely monitored variables particularly with plasma viraemia levels, while cell-associated virus appears to be the major stimulus for sCD163 levels, reflecting a deeper level of virological suppression. Levels of sCD163 also appear to capture the influence of a broad range of CVD risk factors, potentially revealing insights into the inflammatory component of cardiovascular disease that may be particularly relevant in the setting of HIV infection. Lastly, while levels of sCD14 are not associated with routinely monitored variables, this study suggests integrated virus is associated with sCD14, and that cessation of smoking along with the use of integrase inhibitor therapy could significantly reduce levels of this monocyte activation marker, in turn potentially decreasing mortality risk during HIV infection. With the ongoing identification of pathways leading towards monocyte activation, targeting these sources (i.e.ADAM17, IFN-α, TNF-α) could lead to useful therapeutic strategies to reduce immune activation, which has been shown to be associated with the development of age-related disease and mortality in a treated HIV setting. Further research into these mechanisms is warranted, underpinned by the evolution of new HIV monitoring strategies that reflect the underlying chronic inflammatory disease burden and provide insights into pathogenesis and response to treatment.

## Supporting Information

S1 Fig Correlations between plasma biomarkers show strong correlation between sCD163 and CXCL10 (A) and sCD163 and sCD14 (B) while there was no correlation between sCD14 and CXCL10 (C).
(TIF)

S2 Fig The levels of the different plasma biomarkers correlate with ethnicity. CXCL10 was significantly lower in Asian but higher in Indigenous Australians (A), sCD163 was significantly higher in Indigenous Australians (B) while sCD14 was significantly lower in Africans (C).
(TIF)

S1 Table Model 1- Multivariate regression results for CXCL10, sCD163 and sCD14 plasma biomarkers showing significant associations with HIV clinical parameters, CVD risk age, gender, ethnicity and smoking. (DOCX)
