# Supplementary figures and images for: Plasma CXCL10, sCD163 and sCD14 Levels Have Distinct Associations with Antiretroviral Treatment and Cardiovascular Disease Risk Factors
Source: PLoS One. 2016 Jun 29;11(6):e0158169. doi: 10.1371/journal.pone.0158169 (PMC4927121; doi:10.1371/journal.pone.0158169)

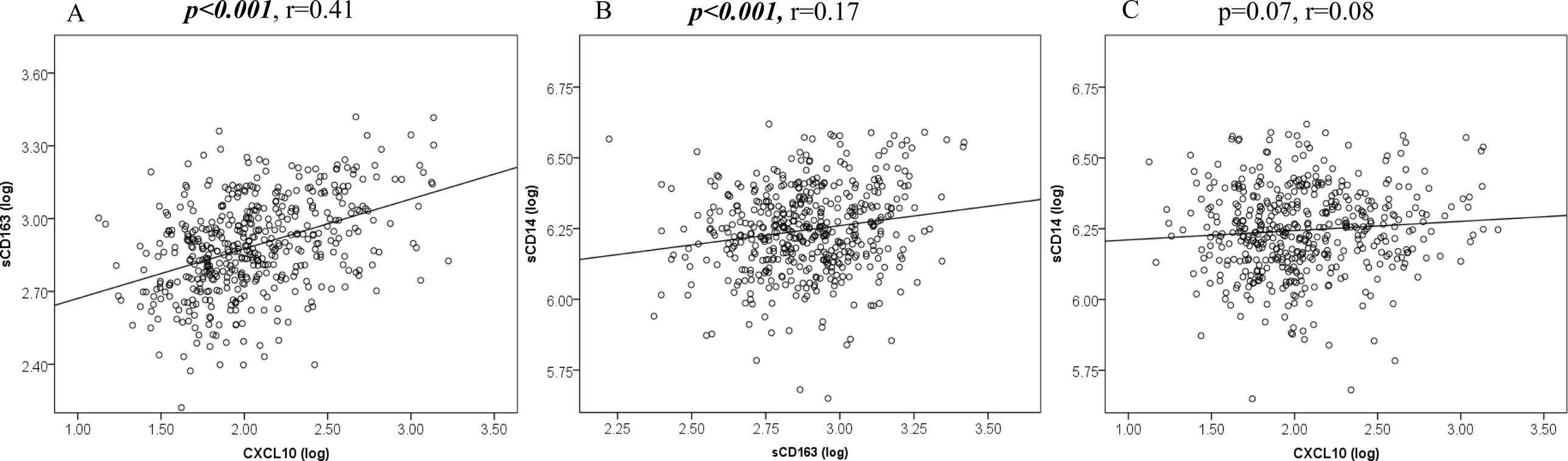

Supplement: S1 Fig — Correlations between plasma biomarkers show strong correlation between sCD163 and CXCL10 (A) and sCD163 and sCD14 (B) while there was no correlation between sCD14 and CXCL10 (C). (TIF) [file pone.0158169.s001.tif]

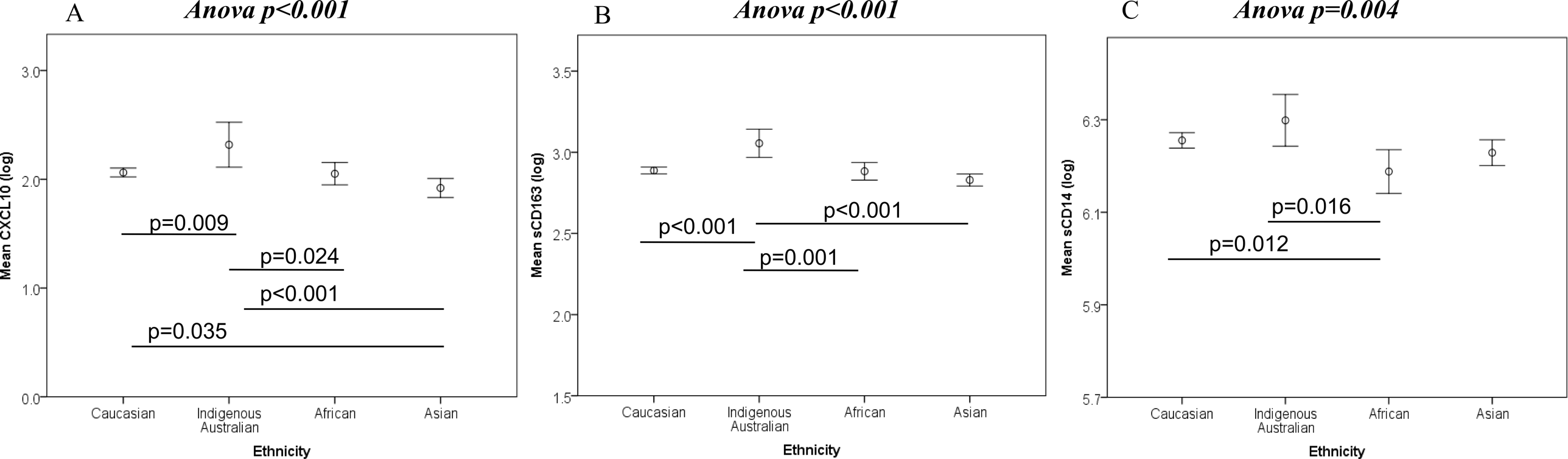

Supplement: S2 Fig — CXCL10 was significantly lower in Asian but higher in Indigenous Australians (A), sCD163 was significantly higher in Indigenous Australians (B) while sCD14 was significantly lower in Africans (C). (TIF) [file pone.0158169.s002.tif]
