# Supplementary material for: Plasma CXCL10, sCD163 and sCD14 Levels Have Distinct Associations with Antiretroviral Treatment and Cardiovascular Disease Risk Factors
Source: PLoS One. 2016 Jun 29;11(6):e0158169. doi: 10.1371/journal.pone.0158169 (PMC4927121; doi:10.1371/journal.pone.0158169)
Supplement: S1 Table — (DOCX) [file pone.0158169.s003.docx]

**S1 Table:** Model 1- Multivariate regression results for CXCL10, sCD163 and sCD14 plasma biomarkers showing significant associations with HIV clinical parameters, CVD risk age, gender, ethnicity and smoking.

| ***Biomarker*** | ***CXCL10*** | | | ***sCD163*** | | | ***sCD14*** | | |
| --- | --- | --- | --- | --- | --- | --- | --- | --- | --- |
| ***Variable*** | **β** | **std error** | **p** | **β** | **std error** | **p** | **β** | **std error** | **p** |
| Age at 2010 | - | - | - | 0.003 | 0.001 | ***0.001*** | 0.001 | 0.0006 | ***0.044*** |
| Gender | - | - | - | - | - | - | 0.035 | 0.018 | 0.057 |
| Ethnicity | -0.18 | 0.047 | ***0.0002*** | 0.116 | 0.045 | ***0.011*** | -0.058 | 0.022 | ***0.009*** |
| Gender:Ethnicity | -0.26 | 0.065 | ***0.0001*** | - | - | - | - | - | - |
| Smoking | - | - | - | - | - | - | 0.046 | 0.015 | ***0.002*** |
| Residual viraemia | - | - | - | 0.062 | 0.027 | ***0.022*** | - | - | - |
| VL3 | 0.08 | 0.043 | 0.056 | - | - | - | - | - | - |
| VL4 | 0.12 | 0.063 | 0.054 | 0.082 | 0.037 | ***0.028*** | - | - | - |
| VL5 | 0.29 | 0.060 | ***<0.0001*** | - | - | - | 0.064 | 0.022 | ***0.003*** |
| SQR CD4:8 | -0.22 | 0.083 | ***0.0085*** | - | - | - | - | - | - |
| SQR CD4 | -0.01 | 0.003 | ***0.0001*** | -0.004 | 0.001 | ***0.011*** | -0.002 | 0.001 | 0.051 |
| NRTI | -0.18 | 0.048 | ***0.0002*** | -0.059 | 0.028 | ***0.039*** | - | - | - |
| NNRTI | - | - | - | - | - | - | 0.047 | 0.018 | ***0.008*** |
| PI | - | - | - | 0.038 | 0.019 | 0.052 | 0.042 | 0.018 | ***0.021*** |
| Integrase | - | - | - | - | - | - | -0.088 | 0.038 | ***0.019*** |
| Cholesterol (log) | -0.37 | 0.158 | ***0.02*** | - | - | - | - | - | - |
| HDL | - | - | - | -0.049 | 0.025 | ***0.048*** | - | - | - |
| BMI | 0.006 | 0.003 | 0.078 | 0.005 | 0.002 | ***0.009*** | - | - | - |
| Framingham score | - | - | - | 0.056 | 0.027 | ***0.038*** | - | - | - |
| ACE Inhibitor | 0.099 | 0.049 | ***0.045*** | - | - | - | - | - | - |

“-“= p>0.1; CVD=cardiovascular disease; VL=Viral load; residual viraemia=<0VL≤1.6; VL3=1.6<VL≤3: VL4= 3<VL≤4; VL5= 4<VL≤6; SQR= squared; BMI=body mass index; NRTI=nucleoside reverse transcriptase inhibitor; NNRTI non nucleoside reverse transcriptase inhibitor; PI= protease inhibitor; HDL: high density lipoprotein; BMI=body mass index; ACE= angiotensin converting-enzyme inhibitor.
